# The Wound Healing and Antibacterial Activity of Five Ethnomedical *Calophyllum inophyllum* Oils: An Alternative Therapeutic Strategy to Treat Infected Wounds

**DOI:** 10.1371/journal.pone.0138602

**Published:** 2015-09-25

**Authors:** Teddy Léguillier, Marylin Lecsö-Bornet, Christelle Lémus, Delphine Rousseau-Ralliard, Nicolas Lebouvier, Edouard Hnawia, Mohammed Nour, William Aalbersberg, Kamelia Ghazi, Phila Raharivelomanana, Patrice Rat

**Affiliations:** 1 Laboratoire Chimie-Toxicologie Analytique et Cellulaire-UMR CNRS COMETE 8638, Université Paris Descartes, Sorbonne Paris Cité, Faculté de Pharmacie, Paris, France; 2 Laboratoire Ecosystème Intestinal, Probiotiques, Antibiotiques-EA 4065, Université Paris Descartes, Sorbonne Paris Cité, Faculté de Pharmacie, Paris, France; 3 Laboratoire de Pharmacognosie-UMR CNRS COMETE 8638, Université Paris Descartes, Sorbonne Paris Cité, Faculté de Pharmacie, Paris, France; 4 INRA, Biologie du Développement et Reproduction-UMR 1198, Jouy-en-Josas, France; 5 Laboratoire Insulaire du Vivant et de l'Environnement-EA 4243, Université de la Nouvelle-Calédonie, Nouméa, Nouvelle Calédonie, France; 6 Institute of Applied Sciences, University of the South Pacific, Laucala Campus, Suva, Fiji; 7 Centre de recherche de BioMécanique et BioIngénierie-CNRS UMR 7338, Université de Technologie de Compiègne, Compiègne, France; 8 Equipe Etude Intégrée des Métabolites Secondaires-UMR 241 EIO, Université de la Polynésie Française, Tahiti, FAA'A, Polynésie Française; Université de Technologie de Compiègne, FRANCE

## Abstract

**Background:**

*Calophyllum inophyllum* L. (Calophyllaceae) is an evergreen tree ethno-medically used along the seashores and islands of the Indian and Pacific Oceans, especially in Polynesia. Oil extracted from the seeds is traditionally used topically to treat a wide range of skin injuries from burn, scar and infected wounds to skin diseases such as dermatosis, urticaria and eczema. However, very few scientific studies reported and quantified the therapeutic properties of *Calophyllum inophyllum* oil (CIO). In this work, five CIO from Indonesia (CIO1), Tahiti (CIO2, 3), Fiji islands (CIO4) and New Caledonia (CIO5) were studied and their cytotoxic, wound healing, and antibacterial properties were presented in order to provide a scientific support to their traditional use and verify their safety.

**Methods:**

The safety of the five CIO was ascertained using the Alamar blue assay on human keratinocyte cells. CIO wound healing properties were determined using the scratch test assay on human keratinocyte cells. CIO-stimulated antibacterial innate immune response was evaluated using ELISA by measuring β defensin-2 release in human derivative macrophage cells. CIO antibacterial activity was tested using oilogramme against twenty aerobic Gram- bacteria species, twenty aerobic Gram+ bacteria species, including a multi-drug resistant *Staphylococcus aureus* strain and two anaerobic Gram+ bacteria species e.g. *Propionibacterium acnes* and *Propionibacterium granulosum*. To detect polarity profile of the components responsible of the antibacterial activity, we performed bioautography against a *Staphylococcus aureus* strain.

**Results:**

Based on Alamar Blue assay, we showed that CIO can be safely used on keratinocyte cells between 2.7% and 11.2% depending on CIO origin. Concerning the healing activity, all the CIO tested accelerated *in vitro* wound closure, the healing factor being 1.3 to 2.1 higher compared to control when keratinocytes were incubated after scratch with CIO at 0.1%. Furthermore, our results showed that CIO exhibit two distinct antibacterial effects: one against Gram+ bacteria by direct inhibition of mitotic growth and another potent effect against Gram- bacteria due to increased release of β-defensin 2 peptide by macrophages. Interestingly, the needed concentrations of CIO to inhibit bacteria growth and to promote wound healing are lower than concentrations exhibiting cytotoxic effects on keratinocyte cells. Finally, we performed bioautography assay against *Staphylococcus aureus* to determine polarity profile of the components responsible for CIO antibacterial activity. Our results showed for the five tested CIO that components responsible of the bacterial growth inhibition are the more polar one on the TLC chromatographic profile and are contained in the resinous fraction of the oil.

**Conclusions:**

This study was conducted to evaluate cytotoxicity, wound healing and antibacterial properties of five CIO traditionally used to treat infected wounds. Using cell and bacteria cultures, we confirmed the pharmacological effects of CIO as wound healing and antimicrobial agent. Moreover, we showed that concentration of CIO needed to exhibit therapeutic effects are lower than concentrations exhibiting cytotoxic effects *in vitro*. For the first time, this study provides support for traditional uses of CIO. These wound healing and antibiotic properties make CIO a valuable candidate to treat infected wounds especially in tropical areas.

## Introduction

Chronic wounds remain a major health problem, particularly in tropical areas where high temperature and humidity promote wound bacterial infections. Increase in bacterial resistance to available antibiotics and poor access to treatments worsen this issue [1]. Efforts are needed to find new antibiotics, easy to produce locally and coming from cheap and renewable sources. Since years, plants have been used in traditional medicine to treat a large range of human diseases [2,3]. Leaves and barks are generally prepared in infusion for internal use and oil extracted from the fruits is rather used topically [4]. Among the natural products extracted from the plants that have demonstrated biological activities, some of them need to receive particular attention as antimicrobial and wound healing agents [5,6]. Indeed, new treatments accelerating wound closure and at the same time preventing infections present a great interest, particularly in tropical area. *Calophyllum inophyllum* is a medium to large evergreen tree presenting elliptical leaves, fragrant white flowers and large round nuts [7,8]. The tree is widely distributed along the coasts of the Indian and Pacific Oceans especially in Melanesia and Polynesia [9,10]. Locally called *Tamanu in* Polynesia, *Tamanou de bord de mer* in New Caledonia, *Dilo* in Fiji, *Nyamplung* in Indonesia this latter is traditionally used notably in the treatment of suppurating wounds [11–15]. The decoction of the leaves is employed to relieve dermatosis, urticaria and eczema [16], the juice of the bark is purgative [10], the first cold pressed seed oil is used in wound healing [13] and to relieve neuropathy associated with leprosy [13,17]. Oil is recommended for all kinds of burns, post-surgical and suppurating wounds, dermatoses, acne, psoriasis, herpes, rheumatism and gonorrhea [10,12,18,19]. Recently, a survey performed in the Marquesas archipelago reported that a preparation based on CIO named *pani temanu* is used to cure skin diseases related to itches, skin allergy, burns and mild wounds [20]. Despite this high therapeutic potential for skin disease, only few studies reported *Calophyllum inophyllum* oil (CIO) pharmacological properties. Pocidalo *et al*. described healing properties of CIO resin on animal experimental burns [21] and Patil *et al*. reported that compounds isolated from CIO possessed strong activity against HIV-1 [22]. In another one, four coumarin derivatives obtained from a crude extract of the nuts were shown to be active against *Staphylococcus aureus* [23]. More recently, Said *et al*. showed that CIO presents both UV-absorption and antioxidant properties [24]. Taken together these wound healing and antibiotic properties make CIO a valuable candidate as an alternative therapeutic strategy to treat infected wounds especially in tropical areas. In this study, five CIO from Indonesia, Tahiti, Fiji islands and New Caledonia were compared for their wound healing and antibacterial activities. Using whole oil extracts on human cells and bacterial cultures, we attempt to ascertain the safety of the studied CIO and to confirm their therapeutic effects.

## Materials and Methods

### Plant materials

Five CIO from Indonesia (CIO1), Tahiti (CIO2, 3), Fiji islands (CIO4) and New Caledonia (CIO5) were compared for their pharmacological properties. The references, geographic origins and characteristics of studied oils are summarized in supplementary material (S1 and S2 Tables). *Calophyllum inophyllum* is not listed as endangered or protected species. In Indonesia, Tahiti and Fiji islands, CIO is commercially exploited and no specific permissions are required for scientific investigation. However, a scientific research authorization is required for exploitation of New Caledonia biological resources. In New Caledonia, nuts were collected under the scientific research authorizations N°2188–2010 granted by Department of Environment of the South Province. Oil production was obtained following the manufacturer's guidelines, briefly: nuts were collected after reaching maturity and fresh almonds were extracted from the nuts. Then, fresh almonds were air dried before performing first cold press before being filtrated and packaged under controlled atmosphere. Refined Olive oil (Sigma-Aldrich) was used as control for all the subsequent experiments.

### CIO fatty acid analysis

Lipids from CIO were extracted with chloroform/methanol (2/1). Once extracted the fatty acids were trans-methylated with Boron trifluoride methanol 7% (Sigma-Aldrich, Saint Quentin Fallavier, France). The methyl esters of plasma fatty acids were analyzed by gas chromatography (Auto Sampling 8410 Gas Chromatograph 3900; Varian, Les Ulis, France) coupled to flame ionization detector (FID) on an Econo-Cap EC-WAX capillary column (30-m, 0.32-mm internal diameter, 0.25-μm Film, ref 19654, ALLTECH Associates Inc, Templemars, France), as described [25]. A standard mixture was used to identify each FA methyl ester in samples, the results were expressed as the abundance of each FA relative to the total FA (%) (S2 Table), and total fatty acid were quantified using heptadecanoic acid (C17:0, margaric acid) as an internal standard.

### Cell culture and incubations

Cells were cultured under standard conditions (moist atmosphere of 5% CO_2_ at 37°C) in Dulbecco’s Minimum Essential Medium for HaCaT human keratinocytes (the cell line was obtained directly from the Cell Lines Service, Batch # 300493–2417) [26] and in RPMI-1640 medium for U937 human leukemic monocytes (the cell line was obtained directly from ATCC® CRL-1593.3^TM^) [27]. Both mediums were supplemented with 10% fetal calf serum (FCS), 2mM L-glutamine, 50 IU/ml penicillin and 50 IU/ml streptomycin.

### Macrophage and HaCaT seeding

U937 cells were differentiated into macrophages using phorbol myristate acetate (Sigma-Aldrich P8139) at 16 ng/ml for 48h [28]. Once attached to the flask bottom, the cells were scraped, counted and seeded in 96-well microplates at a density of 200.000 cells per well (1.10^6^ cells/ml) and kept at 37°C for 24h.

70% confluent HaCat cells were removed by trypsin incubation and seeded in 96-well microplates at a density of 20.000 cells per well (1.10^5^ cells/ml) and kept at 37°C for 24h. The cells were incubated for 15 min with different concentrations of CIO, olive oil (as negative control) or PBS (as cell control), then rinsed in PBS and placed in culture medium at 37°C for a 24h-recovery period before performing tests [29].

### Cell viability assessment using the Alamar Blue assay

Alamar Blue is a cell viability indicator that uses the reducing power of living cells to convert resazurin to the fluorescent molecule, resorufin. Resazurin is a nontoxic, cell permeable compound that is blue in color and virtually non fluorescent. Upon entering cells, resazurin is reduced to resorufin, which produces very bright red fluorescence. Viable cells continuously convert resazurin to resorufin, thereby generating a quantitative measure of viability using spectrofluorimetry. We measured cell viability based on the protocol described by Dutot *et al*. [30]. The cells were incubated with Alamar blue at 0.01mg/ml (Life Technology DAL1035) in 2.5% FCS culture medium at 3700B0030C for 6h before performing fluorescent measurement using Safire microplate reader (Tecan 12901300076, λ_exc_ = 535 nm, λ_em_ = 600 nm).

### Wound healing assay

Wound healing assay was adapted from Buonomo’s method [31,32]. HaCaT cells were seeded into 6-well culture plates in 10% FCS culture medium and kept at 37°C for 72 h. Then, culture medium was removed and confluent cells were rinsed with PBS and incubated during 24h in culture medium without FCS. After 24h, confluent cells were wounded with a pipette tip by manual scratch. Medium was removed, cells were rinsed in PBS and plates were placed on graduated pattern before taking pictures of the wound using a Nikon Coolpix camera. PBS was removed and cells were incubated with CIO, olive oil (as negative control) or PBS (as cell control) for 15 min followed by 24h-recovery period in 2.5% FCS culture medium. At D1 (+24h) each wound was taking in picture using the same method described above. The same three areas per well were compared at D0 and D1 to determine the percentage of wound closure. ImageJ (1.48v) was used to analyze pictures. Briefly, wound areas were delimited using freehand selection tool, then we used measurement tool to edit the data window that lists the area in μm^2^ for each wound.

### ß-defensin 2 release measurement

The release of β-defensin 2 in cell supernatants was determined by ELISA (Phoenix Pharmaceutical: EK-072-37). 24h after CIO exposure or olive oil exposure (as control), cell supernatants were harvested and stored at -20°C until β-defensin 2 measurements. The quantity of released β-defensin 2 was measured according to the manufacturer’s instructions.

### Oilogramme

We adapted the dilution method technique [33] in order to determine the minimal inhibitory concentration (MIC) of vegetable oils. This new technique based on oil/water emulsions and convenient to assess MIC with vegetable oils was named oilogramme.

*Preparation of dishes*: Tubes containing 18ml of Mueller-Hinton agar (Biomérieux) were autoclaved and allowed to cool at 50°C in water bath. 2ml of sterile water, olive oil or CIO were added to the tubes. Tubes containing 2ml of oil were emulsified, and then serially diluted in MH medium and water to obtain emulsified MH medium containing 0.001 to 2% final oil concentrations. Finally, emulsified-oil-containing media were poured on the dish.

*Bacterial strains*: Bacterial organisms used were reference and clinical strains choose mostly among species implicated in skin infections (S3–S6 Tables).

*Inoculum preparation for MIC test*: Strains were culture overnight on Trycase soy agar (Biomérieux) at 37°C. Then, colonies were transferred into Mueller-Hinton broth (Biomérieux) and incubated overnight at 37°C. Overnight cultures of about 1.5 Mc Farland unit (about 2.10^8^ CFU/ml) were used as initial suspensions then, diluted in sterile water as to obtain a concentration of 10^6^−10^7^ CFU/ml. These 10^6^−10^7^ CFU/ml suspensions were transferred in a 96-well culture microplate for inoculation. Inoculation pattern for each bacteria strain was represented in S1 Fig.

*Inoculation and incubation of the medium*: Agar dishes were spot inoculated, using a multipoint inoculator system (Multipointelite: SCAN400), with 1μl suspension containing 10^3^−10^4^ CFU, which is the inoculum usually recommended for MIC determination with the agar dilution method [34]. Inoculation was done from the lowest to the highest concentration plates. Inoculated agar plates were allowed to stand at room temperature until the inoculum spot were completely absorbed and then incubated at 37°C overnight.

*Result interpretation*: MIC represents the lowest concentration of an antimicrobial agent that will inhibit the visible growth of a microorganism after overnight incubation. Here, we considered the presence of more than three colonies per spot as a positive growth.

*For oilogramme assays performed on Propionibacterium strains*: we proceeded as described above except for two steps. We used Wilkins Chalgren agar (Oxoid) and bacteria were incubated at 37°C for 48h in anaerobic conditions before oilogramme reading.

### Bioautographic investigations

Bioautography is a technique used to detect the antibacterial components present in an extract. This technique consists in separating extract components on Thin Layer Chromatography (TLC), pouring agar medium on TLC in bacteria dishes, spraying bacteria suspension on the agar medium and visualizing compounds responsible of bacteria growth inhibition [35,36]. TLC (11 cm x 11 cm) were loaded with 0.5, 1 or 2μl of CIO then allowed for migration using dichloromethane/ethyl acetate (90:10, V/V) as eluent. Then, separated bands where visualized under UV light (254 and 365 nm). The solvent was completely evaporated from the TLC at room temperature for 48h before performing antibacterial tests. In order to determine which band(s) was/were responsible for CIO antibacterial activity, TLC were placed in bacteria dishes and Muller-Hinton agar medium was poured to allow bacterial cultures on solid medium. The inoculum of *Staphylococcus aureus* was spread over the entire surface of the agar plate by swabbing and culture was incubated overnight at 37°C. Then cultures were observed to localize bacterial growth inhibition areas.

### Statistical analysis

All experiments were conducted in triplicates and values are expressed as mean ± standard deviation. CIO were compared to olive oil as control to perform statistical analysis. For all experiments, olive oil has no effect compared to PBS incubation (data not shown) except in the wound healing assay, where olive oil induced a statistically significant effect on wound closure compare to PBS (the percentage of wound closure was 45±3% with olive oil and 26±2% with PBS). Statistical analysis was performed using one way ANOVA and results were compared by Student t-test at a 5% significance level.

## Results

### Determination of CIO nontoxic concentrations for *in vitro* experiments

**Fig 1:**
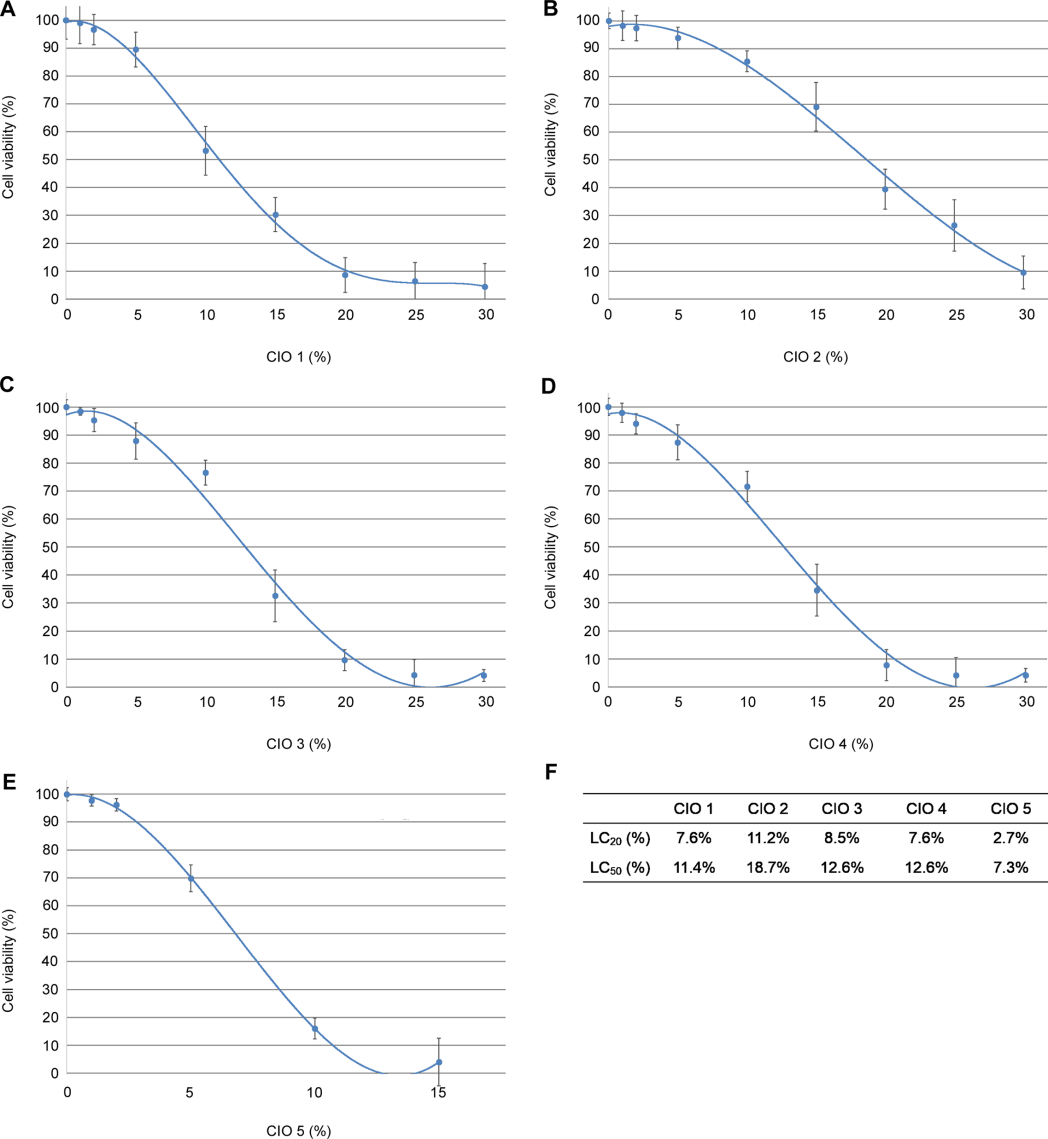
Cytotoxic effect of CIO on a human keratinocyte cell line: HaCaT cells. Cells were treated with various concentrations of CIO (diluted in olive oil) for 15 min followed by 24h-recovery period. Then, cell viability was evaluated using the Alamar Blue assay. Values are expressed as mean ± SD, olive oil was used as control (CIO: 0%). The toxicity of olive oil (as negative control) compared to PBS (as cell control) was not statistically significant (data not shown). (**A-E**) Cell viability after CIO1-5 treatment, (**F**) LC_20_ (%) and LC_50_ (%) were calculated for each CIO using polynomial equations.

Because CIO could contain toxic agents, we first investigated CIO cytotoxicity. We calculated LC_50_ as an indicator of CIO toxicity and LC_20_ as an indicator of CIO maximal nontoxic concentration usable *in vitro*. After 15 min of incubation with CIO at the indicated concentrations (0–30%) and a 24h-recovery period, CIO was found to affect HaCaT cell viability in a concentration-dependent manner (Fig 1A–1E). The percentage of cell viability was calculated based on a comparison with cells incubated with olive oil. Three CIO (Fig 1F) exhibited comparable LC_50_ (CIO1: 11.4%, CIO3 and CIO4:12.6%), CIO2 and CIO5 present the lowest and the highest LC_50_ respectively (CIO2: 18.7% and CIO5:7.3%). These data suggested that the level of cytotoxic compounds was not the same among tested CIO: CIO5 appears as the most cytotoxic, CIO2 as the less cytotoxic and CIO1,3 and CIO4 present an intermediary level of toxicity on HaCaT cells. Based on LC_20_ (CIO1,4: 7.6%, CIO2:11.6%, CIO3: 8.6% and CIO5: 2.7%) as maximal nontoxic concentration usable with HaCaT cells, we decided to study potent CIO therapeutic properties between 0 and 2% in further experiments.

### CIO promotes wound healing in HaCaT keratinocyte cells

**Fig 2:**
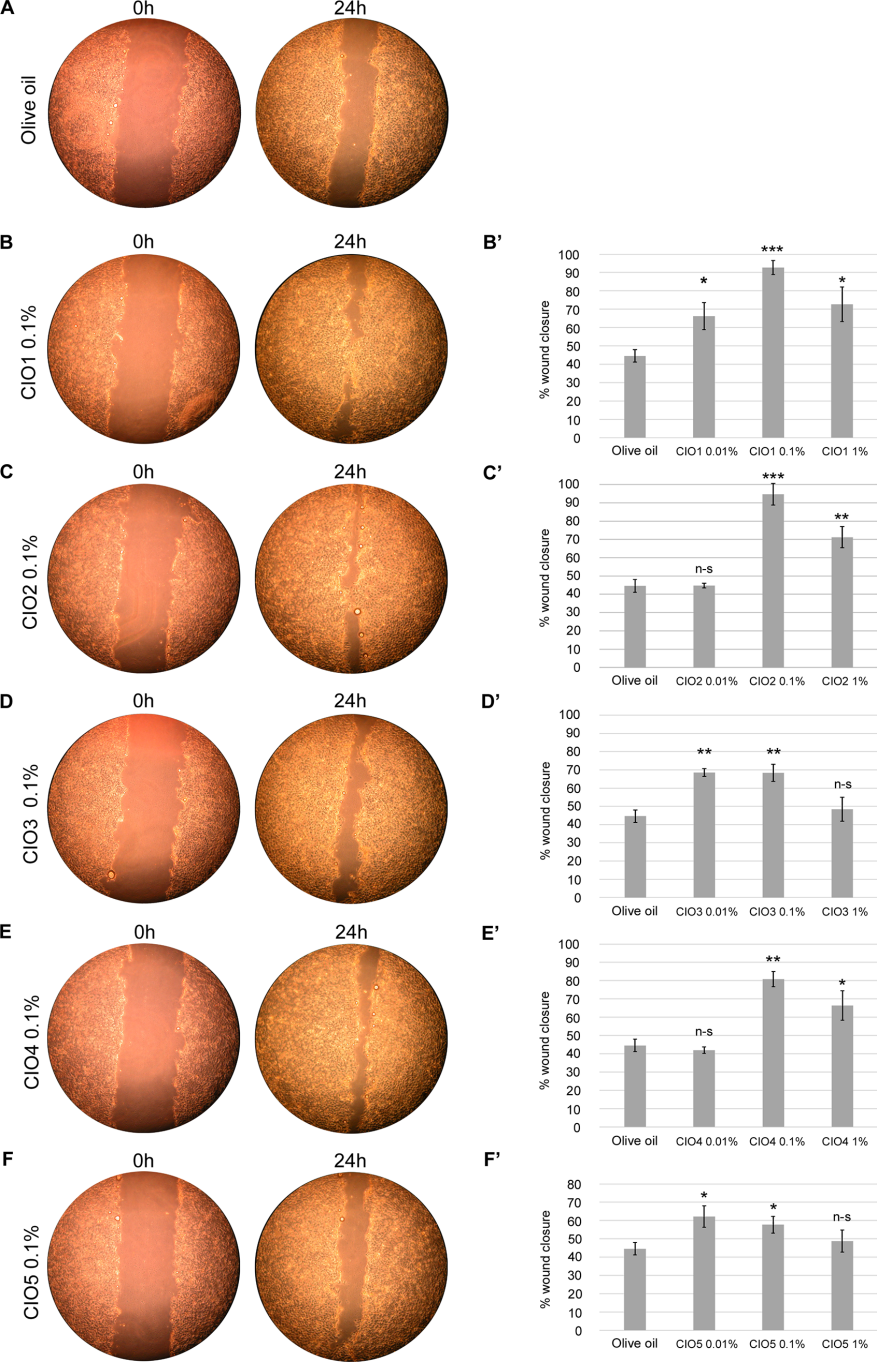
CIO effect on wound healing. Cell migration ability was determined by scratching a confluent HaCaT cell monolayers with a pipette tip. Cells were incubated with olive oil or various concentrations of CIO (diluted in olive oil) for 15 min followed by a 24h-recovery period. Olive oil (as negative control) compared to PBS (as cell control) didn’t exert an inhibitory effect on wound closure (data not shown). Microscopic bright field pictures were taken after the scratch and the precise coordinates were reassessed 24h after incubation. (**A**) Example of scratch assay in olive oil; (**B-F**) Examples of scratch assays at 0.1% CIO1-5; (**B’-F’**) CIO1-5 quantification of wound closure at 0.01, 0.1 and 1% concentration. CIO1-5 were compared to olive oil as control using t-tests. n-s: non-significant, *: 0.01 < p < 0.05, **: 0.01 < p, ***: 0.001 < p.

We evaluated CIO wound healing stimulating activity on HaCaT cells using the scratch assay. Scratches were realized on confluent HaCat cells 24h after CIO exposure at three concentrations (0.01, 0.1 and 1% diluted in olive oil). At 0.1%, the five CIO induced a statistically significant effect on wound closure compared to control (Fig 2). CIO1-4 exhibited their highest wound closure rate at 0.1% (CIO1: 93±4%, CIO2: 95±6%, CIO3: 68±5% and CIO4: 81±4%) and CIO5 exhibited its highest wound closure rate at 0.01% (CIO5: 62±6%) compared to control (Olive oil: 45±3%). These data show that CIO1 and CIO2 exhibit higher activity on keratinocyte wound closure than the other CIO. In all cases, CIO needed concentrations to exhibit healing properties are 27 to 76 lower than concentrations presenting cytotoxic effects on keratinocyte cells, supporting CIO as a safe topical agent.

### CIO increase β-defensin 2 release, an antimicrobial peptide active against Gram- bacteria

**Fig 3:**
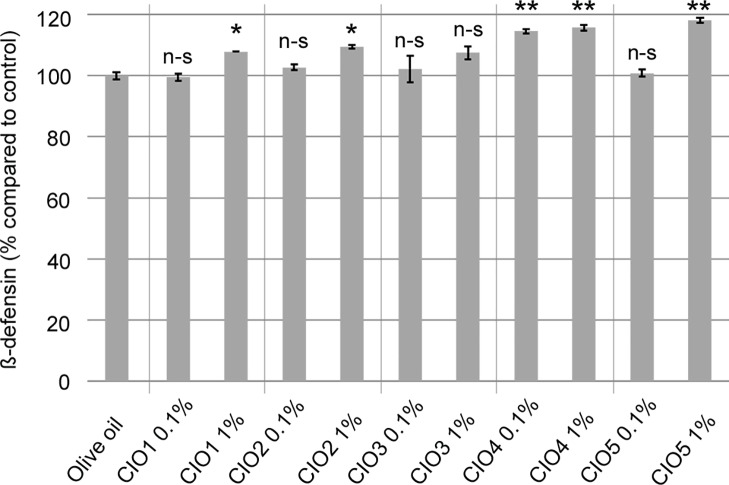
CIO effect on β-defensin 2 release by U937 derivative macrophages. Macrophages were incubated with olive oil as control or with various concentrations of CIO (diluted in olive oil) for 15 min followed by a 24h-recovery period. Cell supernatants were collected 24h after CIO exposure to determine β-defensin 2 release using ELISA. CIO1-5 were compared to olive oil as control using t-tests. n-s: non-significant, *: 0.01 < p < 0.05, **: 0.01 < p.

ß-defensin 2 is an antimicrobial peptide produced by various cells implicated in innate immune response such as macrophages, and is notably active against Gram- bacteria [37]. To investigate CIO implication in innate immune response, we assessed its capacity to induce β-defensin 2 release by U937 derivative macrophage cells. Macrophages were preincubated with CIO at the indicated concentrations for 15 min. Supernatants were collected 24h after CIO exposure to determine β-defensin 2 release. As seen in Fig 3B, at 0.1% CIO4 is the only one to induce a statistically significant increase in β-defensin 2 release compared to control (CIO4: 114.51±0.79%). At 1% (Fig 3B) four of the tested CIO induce a statistically significant increase in β-defensin 2 release compared to control (CIO1: 107.85±0.02%, CIO2: 109.44±0.62%, CIO4: 115.68±1.03%, CIO5: 118.07±0.77%, olive oil: 100.00±0.85%). Our data indicate that CIO4 and CIO5 are the highest inducer of β-defensin 2 release in U937 derivative macrophage cells.

### CIO exhibits high antibacterial activity against bacteria involved in skin infections such as multi-drug resistant *Staphylococcus aureus* strain

**Fig 4:**
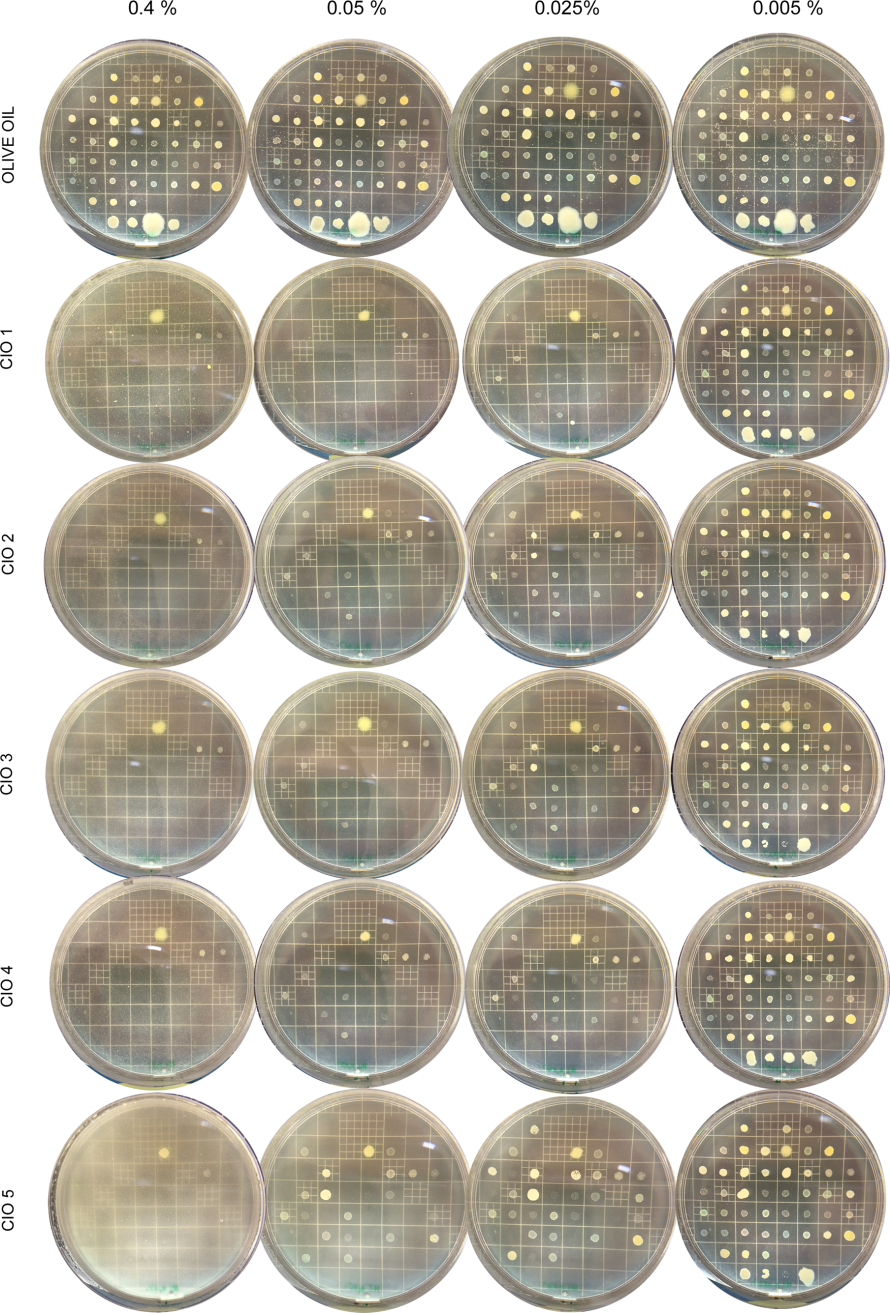
Oilogramme. The dilution method technique was adapted in order to determine the minimal inhibitory concentration (MIC) of the 5 vegetable oils. 49 bacterial strains (aerobic Gram- and Gram+ bacteria) were inoculated at ≈10^3^ colonies per spot on agar medium emulsified with various concentrations of olive oil as control or CIO1-5 (0.001% to 2%). Pictures were taken 24h after inoculation for each oil concentration tested. Only four oil concentrations are represented (0.4%, 0.05%, 0.025% and 0.005%). The presence of at least three visible colonies per spot was considered as positive growth.

**Fig 5:**
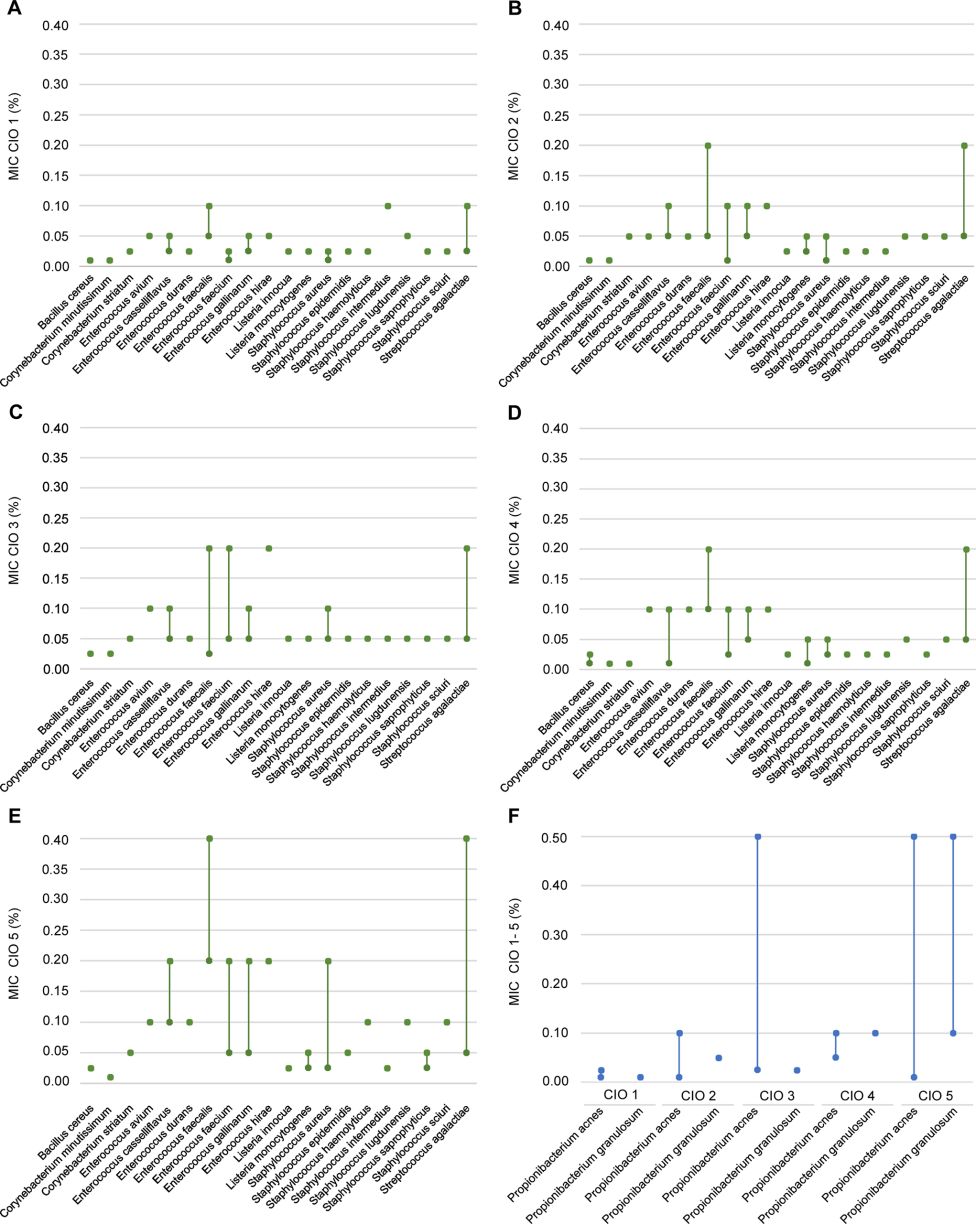
CIO antibacterial activity against Gram+ bacteria. The min and max MIC values observed with oilogramme assay are represented for each bacterial strain and each CIO. For one species, a single spot indicates identical min and max MIC value for each tested strain and two spots linked indicate different min and max MIC values among tested strains (For example we have tested 8 strains for *Staphylococcus aureus* species). (**A-E**) Aerobic Gram+ bacteria MIC value range for CIO1-5. (**F**) Anaerobic Gram+ bacteria MIC value range for CIO1-5.

To evaluate the antibacterial properties of CIO, we adapted the dilution method to determine the Minimal Inhibitory Concentration (MIC) values with vegetable oils; this new technique was named oilogramme. A preliminary screening, using oilogramme with 2% CIO showed no activity against twenty five strains of Gram—bacteria representing twenty species (S3 Table). Then, for further investigations, a panel of forty nine aerobic bacterial strains (forty six Gram+ representing twenty species and three Gram-), mostly implicated in skin diseases, and including multidrug resistant strains, was tested against 0.001 to 2% CIO concentrations using oilogramme (S4 and S5 Tables). Fig 4 shows oilogramme at four oil concentrations (0.4, 0.05, 0.025 and 0.005%), olive oil (used as control) did not inhibit bacterial growth whatever the tested strain. The three tested aerobic Gram- bacteria species were *Achromobacter xylosoxidans denitrificans*, *Achromobacter xylosoxidans xylosoxidans* and *Pseudomonas aeruginosa*. The only bacteria that grew when incubated with 0.4% CIO were the Gram- strains, while none of the Gram+ strains exhibited growth at this concentration. These data suggest that the concentrations of CIO we used here were poorly active against Gram- bacteria. Concerning aerobic Gram+ bacteria, we tested notably *Staphylococcus aureus* largely involved in nosocomial and skin infections, *Bacillus cereus* associated to wound infections in postsurgical patients and cutaneous infections subsequent to trauma, *Staphylococcus epidermidis* and *Staphylococcus haemolyticus* responsible for catheter associated infections and *Corynebacterium minutissimum* implicated in erythrasma. At a concentration of 0.4% all Gram+ bacteria were inhibited in CIO1-5 plates (Fig 5A–5E). These data show that CIO1-5 at 0.4% was active against all studied Gram+ bacteria including a multi-drug resistant *Staphylococcus aureus* strain (S4 and S6 Tables, strain CRBIP21.21). The MIC value profile for Gram+ bacteria (min and max MIC values per strain) for each CIO was represented Fig 5. Gram+ MIC value range was 0.01 to 0.1% for CIO1, 0.01 to 0.2% for CIO2 and CIO4, 0.025 to 0.2% for CIO3 and 0.001 to 0.4% for CIO5 (Fig 5). Our results show that CIO1 is the most active oil against Gram+ bacteria. Moreover, all the tested CIO against Gram+ bacteria species present MIC value similar or lower than ofloxacin (S4 Table) suggesting that CIO could be topically used for prevention or treatment of Gram+ skin infections. It is interesting to note that the tested multi-drug resistant *Staphylococcus aureus* strain is more sensitive to CIO than ofloxacin (0.025% for CIO1 versus 6.400% for ofloxacin) (S4 Table). In this context, CIO appears as a promising source to develop new antibiotics notably to fight multi-drug resistant bacteria implicated in skin infections.

### CIO exhibits high antibacterial activity against bacterial strains involved in acne

We also used the oilogramme to test CIO antibacterial activity against twenty three stains of anaerobic Gram+ bacteria, e.g. *Propionibacterium acnes* and *Propionibacterium granulosum*, both involved in acne (S5 Table). Fig 5F shows that the five tested CIO were highly active against these strains. The MIC value range was 0.01 to 0.025% for CIO1, 0.01 to 0.1% for CIO2, 0.05 to 0.5% for CIO3, 0.05 to 0.1% for CIO4 and 0.01 to 0.5% for CIO5. These results show that CIO1 is the most active oil against both *Propionibacterium* species. In an interesting manner all the tested CIO against *Propionibacterium* species present MIC value similar or lower than ofloxacin (S5 Table). These data strongly suggest that CIO could be topically used in the treatment of acne.

### CIO presents high selectivity index against Gram+ bacteria

**Fig 6:**
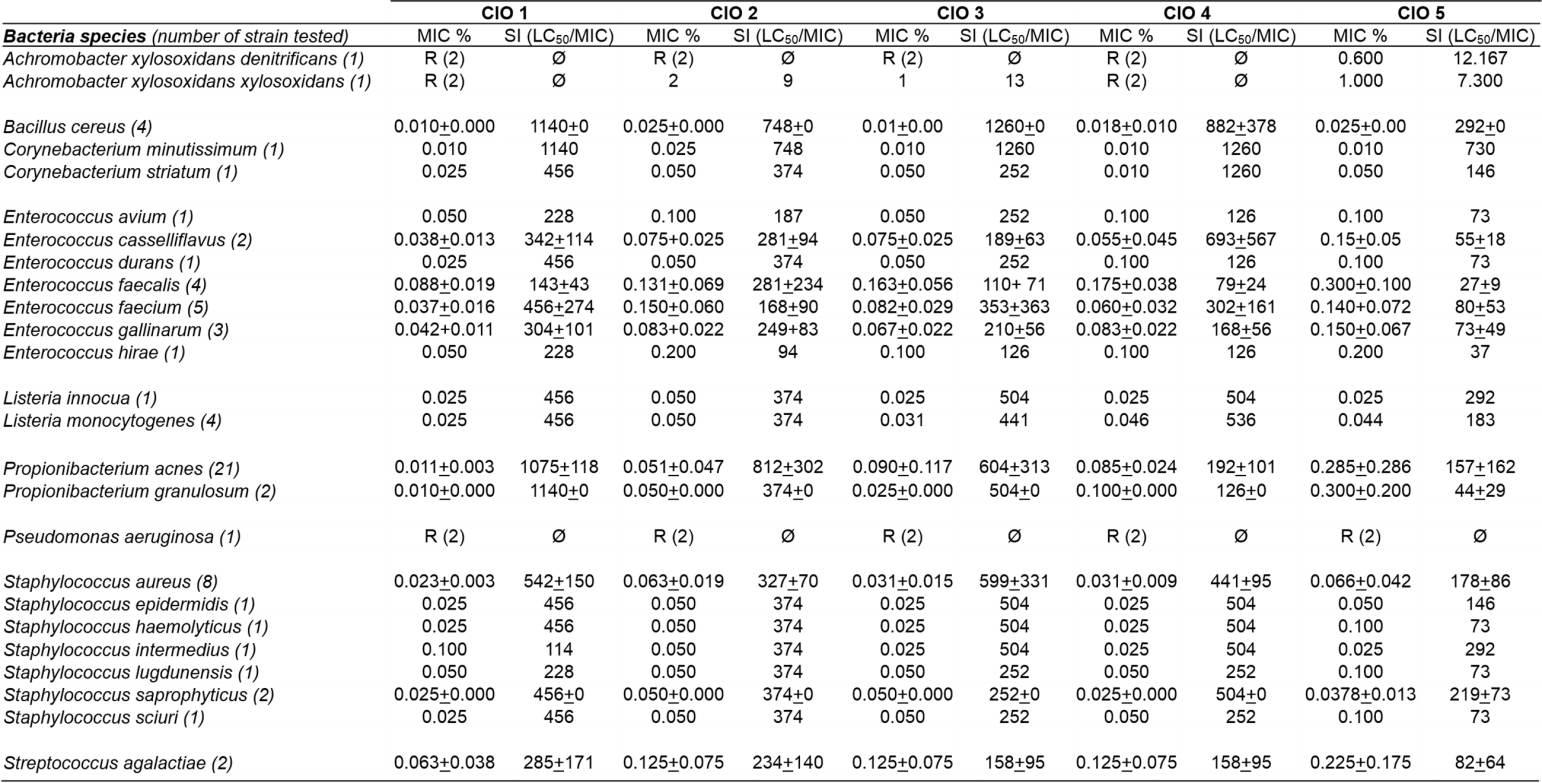
MIC (%) and specific activity index (SI) of CIO1-5 against 25 bacterial strains calculated by dividing cytotoxicity (LC_50_) by MIC.

Finally, we calculated the specific index (SI) also called Therapeutic index for all the bacteria strains tested against CIO1-5 (Fig 6). SI values were calculated by dividing cytotoxicity LC_50_ values by the MIC values in the same units (SI = LC_50_/MIC). SI is considered as a measure of potential efficacy versus adverse effects, i.e. the higher the SI is for an extract, the more likely it is that the activity is not due to a general toxic compound. An SI>1 for an extract increases the likelihood that its toxic activity is not dependent on the antibacterial compounds [38]. For all the tested CIO the SI values were comprised between 73 and 1260. This high therapeutic index value means that CIO may be used for treatment of infections with very low toxicity under controlled conditions.

### Spotting of CIO antibacterial components by bioautography

**Fig 7:**
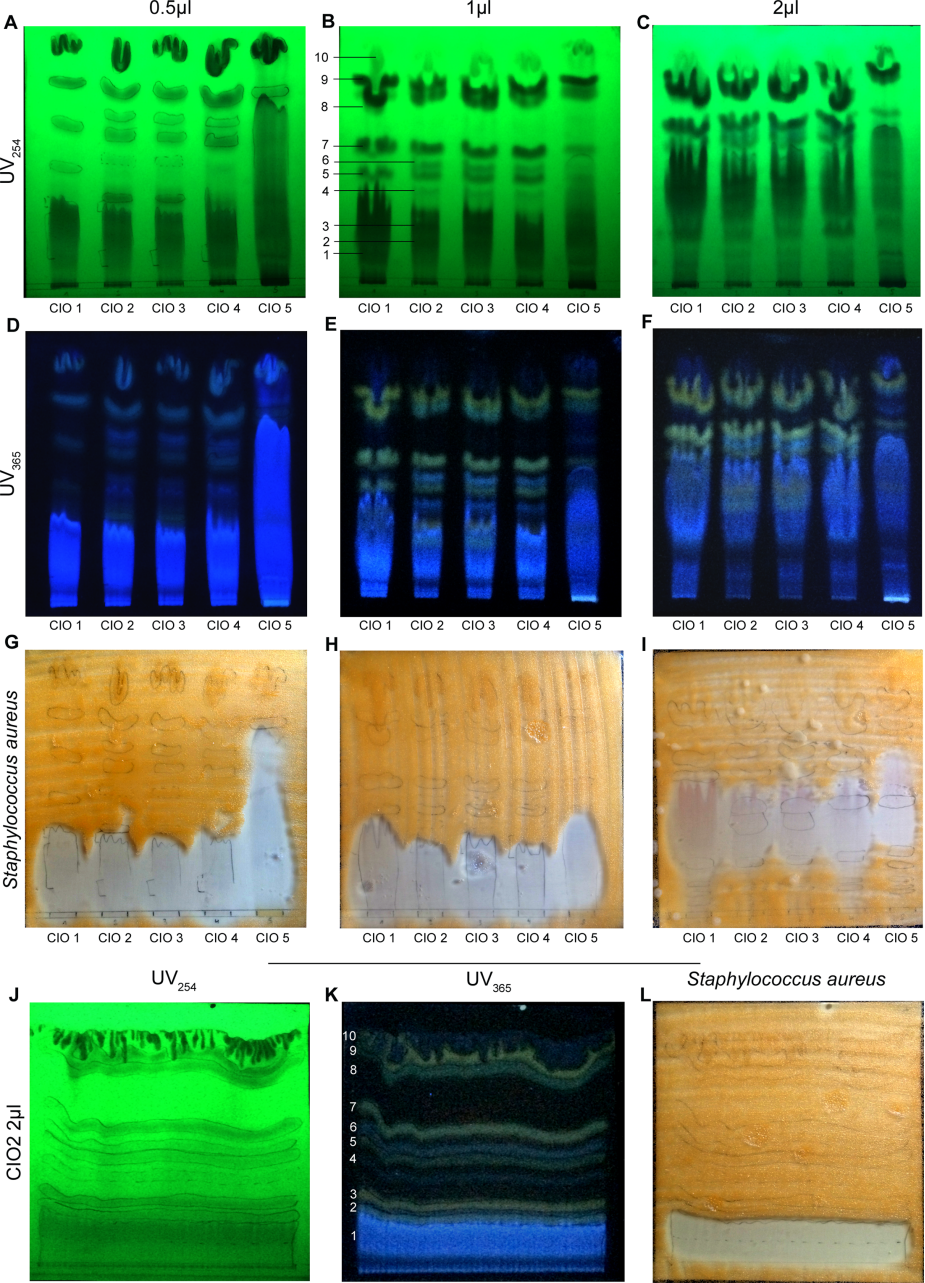
CIO bioautography against *Staphylococcus aureus*. TLC were loaded with 0.5, 1 or 2μl of CIO1-5 before migration in dichloromethane/ethyl acetate (90:10). (**A-F**) Separated bands were then visualized under UV light at 254 and 365 nm. (**G-I**) White areas indicate components that inhibited *Staphylococcus aureus* growth. (**J-K**) TLC loaded with 2μl of CIO2 and visualized under UV light. (**L**) White areas indicate bands that inhibited *Staphylococcus aureus* growth.

To detect the polarity of antibacterial components present in CIO, we used bioautography, which consists in separating CIO compounds on Thin Layer Chromatography (TLC). In a first time, we loaded three TLC with 0.5, 1 and 2μl of CIO1-5. After TLC migration in appropriate solvent, ten major bands were visualized by UV light (Fig 7A–7F). CIO2, CIO3 and CIO4 exhibited similar profiles compare to CIO1 and CIO5 (Fig 7B). This variation observed in CIO composition seems to depend on CIO geographic origin. Then we used *Staphylococcus aureus* (CIP 4.83, S4 Table) culture as bacteria spray to localize bacterial growth inhibition areas. Fig 7G–7I showed that for all the tested CIO the most polar band appearing in blue under UV at 365 nm provided an inhibition of the bacteria growth at 0.5, 1 and 2μl. Because band n°1 overlaps bands n°2 and n°3 we performed a new TLC by loading 2μl of a single CIO to better separate the different components (Fig 7J and 7K). This TLC was used to perform bioautography and Fig 7J–7L showed that band n°1 was indeed responsible for *Staphylococcus aureus* growth inhibition.

### Resin extracted from CIO is responsible of its antibacterial activity

**Fig 8:**
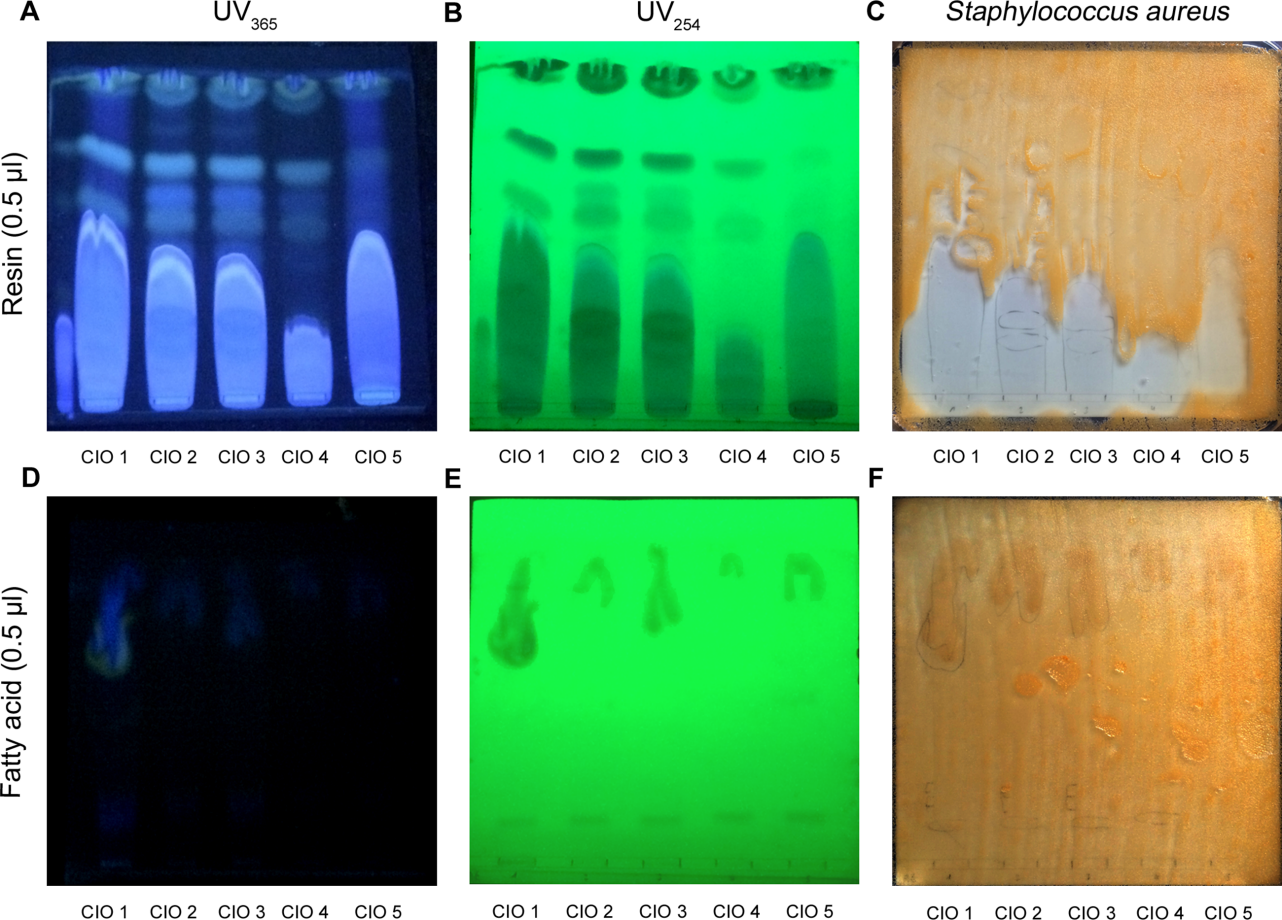
CIO resin and fatty acid bioautography against *Staphylococcus aureus*. TLC were loaded with 0.5μl of CIO1-5 resin (**A-C**) or CIO1-5 fatty acid (**D-F**) before migration in dichloromethane/ethyl acetate (90:10). (**A,B,D,E**) Separated bands were then visualized under UV light at 254 and 365 nm. (**C-F**) White areas indicate components that inhibited *Staphylococcus aureus* growth.

As described by Pocidalo *et al*, CIO healing properties on animal experimental burns depended on its resin (21). We therefore hypothesized that others CIO pharmacological properties such as antibacterial activity could also result from it. Thus, initially, we separate resin from fatty acids of CIO1-5 following extraction process previously described by Petard *et al* (17). Then we loaded two distinct TLC, one with 0.5μl of CIO1-5 resins and the other with 0.5μl of CIO1-5 fatty acids. After migration in appropriate solvent, TLCs were visualized by UV light presented on Fig 8A, 8B, 8D and 8E. We then used *Staphylococcus aureus* (CIP 4.83, S4 Table) culture on TLCs to localize bacterial growth inhibition areas. Fig 8C showed that for all the resins tested, the most polar band appearing in blue under UV at 365 nm provided an inhibition of the bacteria growth while no inhibition of bacteria growth was observed with fatty acids (Fig 8F). This result indicates that antibacterial activities observed for CIO1-5 were due to its resin.

## Discussion

Due to antibiotic resistance increase and poor access to treatment in some tropical areas, new therapeutics accelerating wound closure and at the same time preventing infections present a great interest (1). Efforts are needed to find new antibiotics, easy to produce locally and coming from cheap and renewable sources. Along the seashores and islands of the Indian and Pacific Oceans, CIO is traditionally topically used to treat a wide range of skin injuries from burn and infected wounds to skin disease such as acne and psoriasis. These wound healing and antibiotic properties make CIO a valuable candidate as alternative therapeutic strategy to treat infected wounds especially in tropical areas. Using five CIO that come from Indonesia, Tahiti, Fiji islands and New-Caledonia we attempted to evaluate their cytotoxicity, wound healing and antibacterial properties.

**Fig 9:**
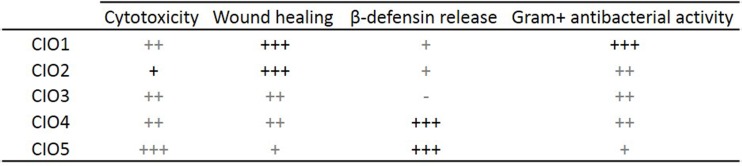
Schematic representation of the pharmacological activities of the five studied CIO.

We first investigated CIO cytotoxicity to determine nontoxic concentrations usable *in vitro*. Then, we investigate CIO wound closure ability on keratinocyte cells. Our results showed that all of the tested CIO accelerate keratinocyte wound healing, CIO1 and CIO2 being the most regenerative oils (Fig 9). These results strengthened the wound healing potential of traditionally used CIO.

As described earlier, other properties of CIO may participate in the wound healing process such as elimination of pathogens in infected wounds. CIO contains palmitic and oleic acids (S2 Table) both known to increase β-defensin 2 production and release [39,40]. This fact should explain that the oily components of CIO such as fatty acids may have another role than being only a natural excipient of bioactive components but also by elicitation of β-defensin. β-defensin 2 is an antimicrobial peptide implicate in skin immunity originally isolated from psoriatic skins [41]. This peptide is produced by various cells implicated in innate immune response such as keratinocyte and macrophage cells and play an important antimicrobial role in host defense against cutaneous pathogens [37]. Interestingly β-defensin 2 presents a dose dependent microbicidal effect starting *in vitro* at concentrations from 1 to 3μM against Gram- bacteria (*Pseudomonas aeruginosa*, *Escherichia coli*) and yeasts (*Candida albicans*) [37]. These data suggest that a small increase in β-defensin 2 release could be sufficient to improve host defense against cutaneous pathogens. We showed that CIO increases β-defensin 2 release with CIO4 and CIO5 being the highest inducers (Fig 9). If this result is confirmed *in vivo*, it would mean that CIO could indirectly participate to the elimination of Gram- bacteria and yeast in skin infections by stimulating innate immune defenses.

In another experiment, we tested CIO capacity to directly inhibit bacteria growth. To address the antibacterial properties of CIO, we used the oilogramme procedure to determine the MIC values with vegetable oils. This new technique based on oil/water emulsion doesn’t require the use of organic solvents such as ethanol or DMSO both known to exhibit cytotoxic effects on living cells [42] which could interfere with MIC determination [43]. Our data showed that CIO1-5 are poorly active against Gram- bacteria strains and highly active against all tested Gram+ bacteria strains with similar or lower MIC values in comparison with ofloxacin (S4 and S5 Tables). Moreover our results indicate that CIO1 is the most active oil against Gram+ bacteria (Fig 9). It has frequently been stated that plant extracts are more active against Gram+ bacteria than against Gram- bacteria. This may be attributed to the cell walls of Gram- bacteria less permeable to antimicrobial compounds [44].

A previous study showed that CIO presents UV-absorption property [24]. In this we have demonstrated CIO wound healing and antibacterial activity against *Propionibacterium acnes* and *Propionibacterium granulosum*, both involved in acne. Acne is one of the most common skin diseases, affecting more than 45 million individuals in the United States. It is estimated that nearly 20 percent of all visits to dermatologists are related to the treatment of acne [45]. Interestingly, zinc topically used in the treatment of acne, presents a MIC value against *Propionibacterium acnes* around of 0.26 to 0.51% [46], whereas the MIC value we observed for CIO1 is around 0.01%. Taken together, CIO activities present a great interest in the treatment of this skin disease and making CIO a valuable candidate to develop new drugs in the treatment of acne.

In recent years, the scientific community underlined that antibacterial drug development was not sufficient to address the problems posed by antibiotic resistance among pathogen bacteria [47]. In our study we showed that compare to ofloxacin CIO are highly active against a multi-drug resistant strain of a species which is largely involved in nosocomial and skin infections: *Staphylococcus aureus*. Furthermore, It is interesting to note that MIC of fucidic acid, topically used for *Staphylococcus aureus* skin infection, is around 0.1% [48], whereas the MIC value observed for CIO1 is around 0.023%. In this context, CIO appears as a promising source to develop new antibiotics notably to fight multi-drug resistant bacteria involved in skin infections.

In order to underscore the components responsible for CIO antibacterial property, we performed bioautography against *Staphylococcus aureus*. Our data showed that in the five tested CIO, the same band was responsible of CIO antibacterial activity. Moreover, we showed that this band was indeed contained specifically in CIO resin and not in the fatty acid part. Our results suggest that the band presenting the inhibition could contain coumarins due to its blue fluorescence under UV [49] and could be in accordance with the antibacterial properties against *Staphylococcus aureus* observed for four coumarins isolated from the nuts of *Calophyllum inophyllum* (calaustralin, calophyllolide, inophyllum C, inophyllum E) [23]. Moreover, coumarins were described as water soluble, blue fluorescent dyes under UV light and they can be qualitatively revealed using Thin Layer Chromatography (TLC) [49]. Then, we calculated the specific index (SI) for all the bacteria strains tested against CIO1-5. For all of them the SI values were higher than 1. These results suggest that CIO could represent a potential safe source of raw medicine material for clinical treatment of wound infections.

In an interesting manner, we have shown that the needed concentrations of CIO to inhibit bacteria growth are lower than the concentrations needed to promote *in vitro* wound healing. These wound healing and antibiotic properties make CIO a valuable candidate in the treatment of infected wounds. Besides we observed differences in the level of CIO pharmacological activity depending on their origin (Fig 9). For example, we showed that wound healing on keratinocyte cells was quite similar in CIO3 and CIO4 but lower in CIO5 and higher in CIO1 and CIO2 (Fig 9) suggesting variations in CIO composition. Indeed, we observed variations of composition revealed on TLC with the five CIO and revealed by fatty acids analysis (S2 Table). These differences could result from oil extraction processes, genetic factors as well as environmental influences. Finding of tamanolides which is a new class of pyranocoumarins from French Polynesian CIO [50], and not yet reported from the other CIO geographical origins supports this suggestion. Chemical variability of leaf bioactive components of *Calophyllum inophyllum* had been also shown at a geographical scale of French Polynesia [51] which may results from the above mentioned factors and should be expected in CIO composition throughout Oceania region.

Further investigations will be needed to establish and understand the variations of CIO composition from different origins and how these variations can influence pharmacological properties of the oil. Moreover, characterization of CIO phytochemical composition will be essential to understand CIO antibacterial and regenerative activities and to further decorticate underneath molecular mechanisms of action.

## Conclusions

This study was conducted to evaluate the cytotoxicity, wound healing and antibacterial properties of five CIO traditionally used to treat infected wounds across Oceania. Using human cell and bacteria cultures, we highlighted the pharmacological effects of CIO, proposed as a wound healing and antimicrobial agent. We observed differences in the pharmacological activities of the CIO tested, depending on their origin and probably their variable compositions. We showed that concentration of CIO needed to exhibit therapeutic effects are lower than concentrations exhibiting cytotoxic effects *in vitro* substantiating CIO for their safe topically use in infected wounds and skin diseases such as acne. For the first time, this study provides support for traditional uses of CIO in the wound healing process particularly for infected wounds.

## Supporting Information

S1 Fig Aerobic Gram-negative and Gram-positive bacteria repartition on the plates. Each number correspond to bacteria species described in S3 Table.
(PDF)

S1 Table References, geographic origins and characteristics of CIO. (PDF)

S2 Table Fatty acid composition of CIO. (PDF)

S3 Table Gram-negative bacterial strains tested in preliminary assay. (PDF)

S4 Table Aerobic Gram-negative and Gram-positive bacteria tested against CIO. (PDF)

S5 Table Anaerobic Gram-positive bacteria tested against CIO. (PDF)

S6 Table Bacterial strains resistant to antibiotics. (PDF)
