# Supplementary material for: The Wound Healing and Antibacterial Activity of Five Ethnomedical Calophyllum inophyllum Oils: An Alternative Therapeutic Strategy to Treat Infected Wounds
Source: PLoS One. 2015 Sep 25;10(9):e0138602. doi: 10.1371/journal.pone.0138602 (PMC4583440; doi:10.1371/journal.pone.0138602)
Supplement: S1 Fig — Each number correspond to bacteria species described in S3 Table. (PDF) [file pone.0138602.s001.pdf]

**S1 Figure. Aerobic Gram-negative and Gram-positive bacteria repartition on the plates. Each number correspond to bacteria species described in Table S2.**

| | | | | | | | |
|---|---|---|---|---|---|---|---|
| | | 1 | 2 | 3 | 4 | | |
| | 5 | 6 | 7 | 8 | 9 | 10 | |
| 11 | 12 | 13 | 14 | 15 | 16 | 17 | 18 |
| 19 | 20 | 21 | 22 | 23 | 24 | 25 | 26 |
| 27 | 28 | 29 | 30 | 31 | 32 | 33 | 34 |
| 35 | 36 | 37 | 38 | 39 | 40 | 41 | 42 |
| | 43 | 44 | 45 | 46 | 47 | 48 | |
| | | 49 | 50 | 51 | 52 | | |
