# Supplementary material for: The Wound Healing and Antibacterial Activity of Five Ethnomedical Calophyllum inophyllum Oils: An Alternative Therapeutic Strategy to Treat Infected Wounds
Source: PLoS One. 2015 Sep 25;10(9):e0138602. doi: 10.1371/journal.pone.0138602 (PMC4583440; doi:10.1371/journal.pone.0138602)
Supplement: S1 Table — (PDF) [file pone.0138602.s002.pdf]

S1 Table. References, geographic origins and characteristics of CIO

Name	Reference	Origin	Color	Specific gravity, 20°C
CIO 1	Soliance, batch #J-11	Indonesia	yellow	0.90
CIO 2	Manuia-tamanu, batch # 2014	Tahiti	green	0.89
CIO 3	Pacifique-sud, batch #140270 HTAM (5ek)	Tahiti	green	0.91
CIO 4	Island Naturals batch #1	Fiji islands	green	0.90
CIO 5	Distillerie de Boulouparis, batch #2010	New Caledonia	brown	0.93
