# Supplementary material for: The Wound Healing and Antibacterial Activity of Five Ethnomedical Calophyllum inophyllum Oils: An Alternative Therapeutic Strategy to Treat Infected Wounds
Source: PLoS One. 2015 Sep 25;10(9):e0138602. doi: 10.1371/journal.pone.0138602 (PMC4583440; doi:10.1371/journal.pone.0138602)
Supplement: S2 Table — (PDF) [file pone.0138602.s003.pdf]

S2 Table. Fatty acid composition of CIO

Fatty acids (%)	Fatty acids	Olive oil	CIO 1	CIO 2	CIO 3	CIO 4	CIO 5
**Palmitic acid**	**C16:0**	**16.69**	**15.89**	**15.49**	**16.43**	**16.60**	**14.87**
Palmitoleic acid	C16:1 w7	1.45	0.27	0.61	0.28	0.25	0.15
**Stearic acid**	**C18:0**	**27.19**	**23.72**	**28.62**	**29.14**	**17.69**	**23.73**
**Oleic acid**	**C18:1 w9**	**43.85**	**28.82**	**28.07**	**28.24**	**39.24**	**30.34**
**Linoleic acid**	**C18:2 w6 gamma**	**9.10**	**29.40**	**23.87**	**24.27**	**24.30**	**27.08**
Gamma-linolenic acid	C18:3 w6 gamma	0.16	0.16	0.24	0.05	0.10	0.11
Alpha-linolenic acid	**C18:3 w3 alpha**	**0.02**	**0.24**	**0.23**	**0.26**	**0.22**	**0.30**
Arachidonic acid	C20:0	0.30	0.62	0.67	0.64	0.57	0.71
Gadoleic acid	C20:1 w9	0.23	0.21	0.20	0.18	0.20	0.00
Gadoleic acid	C20:1 w7	0.13	0.10	0.09	0.09	0.11	0.36
Dihomo-gamma-linolenic acid	C20:3 w6	0.01	0.01	0.01	0.01	0.01	0.02
Behenic acid	C22:0	0.05	0.12	0.18	0.12	0.10	0.33
Docosadienoic acid	C22:2 w6	0.33	0.07	0.01	0.07	0.17	0.05
Saturated Fatty Acid	SFA	44.32	40.49	45.08	46.43	35.12	40.78
Monounsaturated Fatty Acid	MUFA	46.00	29.54	30.48	28.89	40.02	31.44
Polyunsaturated Fatty Acid	PUFA	9.68	29.97	25.37	24.68	24.85	27.83
Polyunsaturated Fatty Acid n-6	PUFA n-6	9.60	29.67	24.14	24.41	24.60	27.44
Polyunsaturated Fatty Acid n-3	PUFA n-3	0.07	0.30	0.30	0.27	0.25	0.34
**Fatty acids (mg)/g of oil**		344.63	296.26	419.61	333.38	320.27	520.92
