# Supplementary material for: The Wound Healing and Antibacterial Activity of Five Ethnomedical Calophyllum inophyllum Oils: An Alternative Therapeutic Strategy to Treat Infected Wounds
Source: PLoS One. 2015 Sep 25;10(9):e0138602. doi: 10.1371/journal.pone.0138602 (PMC4583440; doi:10.1371/journal.pone.0138602)
Supplement: S3 Table — (PDF) [file pone.0138602.s004.pdf]

**S3 Table. Gram –negative bacterial strains tested in preliminary assay.**

| ***Species*** | **Reference** |
|---|---|
| *Achromobacter xylosoxidans denitrificans* | CIP 71.32 |
| *Achromobacter xylosoxidans xylosoxidans* | CIP 77.15 |
| *Acinetobacter baumannii* | CIP 70.34 |
| *Aeromonas hydrophila* | CIP 76.14 |
| *Burkholderia cepacia* | CIP 80.24 |
| *Citrobacter freundii* | CIP 57.32 |
| *Comamonas acidovorans* | N331 |
| *Comamonas testosteroni* | N070 |
| *Enterobacter aerogenes* | CIP 6086T |
| *Enterobacter cloacae* | CIP 60.85 |
| *Escherichia coli* | ATCC25922 |
| *Escherichia coli* | CIP 54.8 |
| *Escherichia coli* | CIP 2.83 |
| *Escherichia coli* | CIP 53126 |
| *Escherichia coli* | CIP 54127 |
| *Klebsiella oxytoca* | E282 |
| *Klebsiella pneumoniae* | CIP 52.145 |
| *Morganella morganii* | E342 |
| *Providencia stuartii* | CIP 107.808 |
| *Pseudomonas aeruginosa* | CIP 103.467 |
| *Pseudomonas aeruginosa* | CIP A22 |
| *Pseudomonas putida* | CIP 55.191 |
| *Salmonella enterica* | CIP 58.58 |
| *Stenotrophomonas maltophilia* | CIP 54.90 |
| *Yersinia enterocolitica* | CIP 80.27 |

References E... and N… : collection of the Laboratoire Ecosystème Intestinal, Probiotiques, Antibiotiques, Faculté de Pharmacie Université Paris Descartes.
