# Supplementary material for: The Wound Healing and Antibacterial Activity of Five Ethnomedical Calophyllum inophyllum Oils: An Alternative Therapeutic Strategy to Treat Infected Wounds
Source: PLoS One. 2015 Sep 25;10(9):e0138602. doi: 10.1371/journal.pone.0138602 (PMC4583440; doi:10.1371/journal.pone.0138602)
Supplement: S4 Table — (PDF) [file pone.0138602.s005.pdf]

S4 Table. Aerobic Gram-negative and Gram-positive bacteria tested against CIO

Spot n°	*Species*	Reference	Mean of olive oil MIC (%)	Mean of CIO1 MIC (%)	Mean of CIO2 MIC (%)	Mean of CIO3 MIC (%)	Mean of CIO4 MIC (%)	Mean of CIO5 MIC (%)	Mean of Ofloxacin MIC (%)
1	*Staphylococcus aureus*	CIP 5710	R (2)	0.025	0.050	0.050	0.025	0.050	0.025
2	*Corynebacterium minutissimum*	E002	R (2)	0.010	0.025	0.010	0.010	0.010	0.025
3	*Staphylococcus aureus*	ATCC 9144 oxford	R (2)	0.025	0.050	0.025	0.025	0.025	0.012
4	*Listeria innocua*	E0044	R (2)	0.025	0.050	0.025	0.025	0.025	0.200
5	*Streptococcus agalactiae*	CIP 103.227	R (2)	0.100	0.200	0.200	0.200	0.400	>12.8
6	*Staphylococcus aureus*	CIP 53156	R (2)	0.025	0.050	0.025	0.025	0.050	0.025
7	*Staphylococcus aureus*	CIP 53154	R (2)	0.025	0.050	0.010	0.025	0.025	0.012
8	*Pseudomonas aeruginosa*	CIP A22	R (2)	R (2)	R (2)	R (2)	R (2)	R (2)	0.100
9	*Enterococcus hirae*	CIP 5855	R (2)	0.050	0.200	0.100	0.100	0.200	0.200
10	*Staphylococcus aureus*	CIP 4.83	R (2)	0.010	0.050	0.010	0.025	0.025	0.025
11	*Staphylococcus saprophyticus*	E260	R (2)	0.025	0.050	0.050	0.025	0.050	0.100
12	*Staphylococcus lugdunensis*	ATCC 43.809	R (2)	0.050	0.050	0.050	0.050	0.100	0.100
13	*Staphylococcus aureus*	ATCC 25.923	R (2)	0.025	0.100	0.025	0.050	0.050	0.050
14	*Staphylococcus epidermidis*	CIP 53.124	R (2)	0.025	0.050	0.025	0.025	0.050	0.050
15	*Staphylococcus haemolyticus*	CIP 81.56	R (2)	0.025	0.050	0.025	0.025	0.100	0.025
16	*Enterococcus faecium*	CIP 103.014	R (2)	0.050	0.200	0.100	0.100	0.200	0.800
17	*Achromobacter xylosoxidans xylosoxidans*	CIP 77.15	R (2)	R (2)	R (2)	R (2)	R (2)	1.000	0.200
18	*Achromobacter xylosoxidans denitrificans*	CIP 71.32	R (2)	R (2)	2.000	1.000	R (2)	0.600	3.200
19	*Streptococcus agalactiae*	CIP 103.227	R (2)	0.025	0.050	0.050	0.050	0.050	0.100
20	*Enterococcus faecalis*	CIP 103.214	R (2)	0.100	0.200	0.200	0.200	0.400	0.200
21	*Staphylococcus aureus*	CIP 7625	R (2)	0.025	0.100	0.050	0.050	0.200	0.050
22	*Enterococcus gallinarum*	N489	R (2)	0.025	0.050	0.050	0.050	0.050	0.200
23	*Enterococcus avium*	CIP 104 053	R (2)	0.050	0.100	0.050	0.100	0.100	0.200
24	*Enterococcus casselliflavus*	CIP 103.018	R (2)	0.050	0.100	0.100	0.100	0.200	0.400
25	*Listeria monocytogenes*	CIP 103.575	R (2)	0.025	0.050	0.050	0.050	0.050	0.200
26	*Staphylococcus saprophyticus*	CIP 76125	R (2)	0.025	0.050	0.050	0.025	0.025	0.100

References E... and N… : collection of the Laboratoire Ecosystème Intestinal, Probiotiques, Antibiotiques, Faculté de Pharmacie Université Paris Descartes.

S4 Table. Aerobic Gram-negative and Gram-positive bacteria tested against CIO

Spot n°	*Species*	Reference	Mean of olive oil MIC (%)	Mean of CIO1 MIC (%)	Mean of CIO2 MIC (%)	Mean of CIO3 MIC (%)	Mean of IO4 MIC (%)	Mean of CIO5 MIC (%)	Mean of Ofloxacin MIC (%)
27	*Enterococcus faecalis*	CIP 104 676	R (2)	0.100	0.200	0.200	0.200	0.400	0.100
28	*Enterococcus gallinarum*	CIP 105 985	R (2)	0.050	0.100	0.100	0.100	0.200	0.400
29	*Enterococcus durans*	CIP 104 999	R (2)	0.025	0.050	0.050	0.100	0.100	0.200
30	*Enterococcus faecium*	N507	R (2)	0.025	0.200	0.100	0.050	0.200	>12.8
31	*Enterococcus casselliflavus*	N487	R (2)	0.025	0.050	0.050	0.010	0.100	0.400
32	*Enterococcus faecium*	N490	R (2)	0.010	0.050	0.010	0.025	0.050	0.400
33	*Enterococcus faecalis*	N491	R (2)	0.050	0.100	0.050	0.100	0.200	0.400
34	*Enterococcus gallinarum*	N492	R (2)	0.050	0.100	0.050	0.100	0.200	0.400
35	*Listeria monocytogenes*	N836	R (2)	0.025	0.050	0.025	0.010	0.050	0.200
36	*Listeria monocytogenes*	N851	R (2)	0.025	0.050	0.025	0.100	0.025	0.200
37	*Enterococcus faecium*	CIP 107.387	R (2)	0.050	0.200	0.100	0.100	0.200	12.800
38	*Enterococcus faecium*	N733	R (2)	0.050	0.200	0.100	0.025	0.050	>12.8
39	*Listeria monocytogenes*	N783	R (2)	0.025	0.050	0.025	0.025	0.050	0.200
40	*Enterococcus faecium*	N823	R (2)	0.050	0.100	0.100	0.100	0.200	>12.8
41	*Staphylococcus intermedius*	N987	R (2)	0.100	0.050	0.025	0.025	0.025	1.600
42	*Staphylococcus sciuri*	N993	R (2)	0.025	0.050	0.050	0.050	0.100	0.100
43	*Staphylococcus aureus*	CRBIP 21.21	R (2)	0.025	0.050	0.050	0.025	0.100	6.400
44	*Corynebacterium striatum*	N840	R (2)	0.025	0.050	0.050	0.010	0.050	>12.8
45	*Enterococcus faecalis*	N518	R (2)	0.100	0.025	0.200	0.200	0.200	0.400
49	*Bacillus cereus*	N349	R (2)	0.010	0.025	0.010	0.025	0.025	0.050
50	*Bacillus cereus*	N258	R (2)	0.010	0.025	0.010	0.010	0.025	0.012
51	*Bacillus cereus*	N190	R (2)	0.010	0.025	0.010	0.010	0.025	0.025
52	*Bacillus cereus*	CIP 6624	R (2)	0.010	0.025	0.010	0.025	0.025	0.025

References E... and N… : collection of the Laboratoire Ecosystème Intestinal, Probiotiques, Antibiotiques, Faculté de Pharmacie Université Paris Descartes.
