# Supplementary material for: The Wound Healing and Antibacterial Activity of Five Ethnomedical Calophyllum inophyllum Oils: An Alternative Therapeutic Strategy to Treat Infected Wounds
Source: PLoS One. 2015 Sep 25;10(9):e0138602. doi: 10.1371/journal.pone.0138602 (PMC4583440; doi:10.1371/journal.pone.0138602)
Supplement: S5 Table — (PDF) [file pone.0138602.s006.pdf]

S5 Table. Anaerobic Gram-positive bacteria tested against CIO

Spot n°	*Species*	Reference	Mean of Olive oil MIC (%)	Mean of CIO1 MIC (%)	Mean of CIO2 MIC (%)	Mean of IO3 MIC (%)	Mean of CIO4 MIC (%)	Mean of CIO5 MIC (%)	Mean of Ofloxacin MIC (%)
1	*Propionibacterium granulosum*	N950	R(2)	0.010	0.050	0.025	0.100	0.500	0.100
2	*Propionibacterium acnes*	N930	R(2)	0.010	0.010	0.025	0.050	0.010	0.100
3	*Propionibacterium acnes*	N929	R(2)	0.010	0.025	0.025	0.100	0.010	0.100
4	*Propionibacterium acnes*	N931	R(2)	0.025	0.500	0.500	0.100	0.500	0.100
5	*Propionibacterium acnes*	N877	R(2)	0.010	0.025	0.500	0.050	0.500	0.100
6	*Propionibacterium acnes*	N878	R(2)	0.025	0.100	0.500	0.100	2.000	0.100
7	*Propionibacterium acnes*	N928	R(2)	0.010	0.025	0.025	0.100	0.100	0.100
8	*Propionibacterium granulosum*	N880	R(2)	0.010	0.050	0.025	0.100	0.100	0.100
9	*Propionibacterium acnes*	N881	R(2)	0.010	0.025	0.025	0.100	0.500	0.100
10	*Propionibacterium acnes*	N882	R(2)	0.010	0.025	0.025	0.100	0.100	0.100
11	*Propionibacterium acnes*	N927	R(2)	0.010	0.025	0.010	0.100	0.050	0.100
12	*Propionibacterium acnes*	N926	R(2)	0.010	0.010	0.010	0.025	0.050	0.100
13	*Propionibacterium acnes*	N895	R(2)	0.010	0.025	0.025	0.100	0.100	0.050
14	*Propionibacterium acnes*	N896	R(2)	0.010	0.025	0.025	0.100	0.100	0.100
15	*Propionibacterium acnes*	N897	R(2)	0.010	0.010	0.010	0.100	0.500	0.100
16	*Propionibacterium acnes*	N898	R(2)	0.010	0.025	0.010	0.025	0.050	0.100
17	*Propionibacterium acnes*	N899	R(2)	0.010	0.025	0.025	0.100	0.100	0.100
18	*Propionibacterium acnes*	N907	R(2)	0.010	0.025	0.025	0.025	0.010	0.100
19	*Propionibacterium acnes*	N908	R(2)	0.010	0.025	0.025	0.100	0.100	0.100
20	*Propionibacterium acnes*	N914	R(2)	0.010	0.025	0.010	0.100	0.100	0.100
21	*Propionibacterium acnes*	N924	R(2)	0.010	0.025	0.025	0.100	0.100	0.100
22	*Propionibacterium acnes*	N925	R(2)	0.010	0.050	0.050	0.100	0.500	0.100
23	*Propionibacterium acnes*	CIP 53 117	R(2)	0.010	0.050	0.025	0.100	0.500	0.050

References E... and N… : collection of the Laboratoire Ecosystème Intestinal, Probiotiques, Antibiotiques, Faculté de Pharmacie Université Paris Descartes.
