# Supplementary material for: The Wound Healing and Antibacterial Activity of Five Ethnomedical Calophyllum inophyllum Oils: An Alternative Therapeutic Strategy to Treat Infected Wounds
Source: PLoS One. 2015 Sep 25;10(9):e0138602. doi: 10.1371/journal.pone.0138602 (PMC4583440; doi:10.1371/journal.pone.0138602)
Supplement: S6 Table — (PDF) [file pone.0138602.s007.pdf]

S6 Table. Bacterial strains resistant to antibiotics

Spot n°	*Species*	Reference	Noticeable resistances
22	*Enterococcus gallinarum*	N489	vancomycin
27	*Enterococcus faecalis*	CIP 104 676	aminoglycosides, chloramphenicol, vancomycin
28	*Enterococcus gallinarum*	CIP 105 985	vancomycin
30	*Enterococcus faecium*	N507	amoxicillin, aminoglycosides, erythromycin, lincomycin, cotrimoxazole
32	*Enterococcus faecium*	N490	amoxicillin, vancomycin
33	*Enterococcus faecalis*	N491	vancomycin
34	*Enterococcus gallinarum*	N492	vancomycin
37	*Enterococcus faecium*	CIP 107.387	amoxicillin, vancomycin, teicoplanin
38	*Enterococcus faecium*	N733	amoxicillin, vancomycin, teicoplanin
40	*Enterococcus faecium*	N823	amoxicillin, vancomycin, teicoplanin
43	*Staphylococcus aureus*	CRBIP 21.21	methicillin, tetracyclin, aminoglycosides, macrolides, lincosamides, streptogramin B, sulfonamides, ciprofloxacin, rifampicin, fosfomycin

References E... and N… : collection of the Laboratoire Ecosystème Intestinal, Probiotiques, Antibiotiques, Faculté de Pharmacie Université Paris Descartes.
